# Exploration and identification of anoikis-related genes in polycythemia vera

**DOI:** 10.3389/fgene.2023.1139351

**Published:** 2023-02-17

**Authors:** Wufuer Aini, Limin Xie, Wanyu Hu, Yuan Tang, Hongling Peng, Guangsen Zhang, Tuo Deng

**Affiliations:** ^1^ National Clinical Research Center for Metabolic Diseases, and Department of Metabolism and Endocrinology, The Second Xiangya Hospital of Central South University, Changsha, Hunan, China; ^2^ Key Laboratory of Diabetes Immunology, Ministry of Education, and Metabolic Syndrome Research Center, The Second Xiangya Hospital of Central South University, Changsha, Hunan, China; ^3^ Clinical Immunology Center, The Second Xiangya Hospital of Central South University, Changsha, Hunan, China; ^4^ Department of Hematology, The Second Xiangya Hospital of Central South University, Changsha, Hunan, China; ^5^ Institute of Molecular Hematopathy, The Second Xiangya Hospital of Central South University, Changsha, Hunan, China; ^6^ Hunan Key Laboratory of Tumor Models and Individualized Medicine, Changsha, Hunan, China

**Keywords:** myeloproliferative neoplasms, anoikis-related genes, differentially expressed genes, protein-protein interaction, hub genes

## Abstract

**Background:** Polycythemia Vera (PV) is a type of typical Myeloproliferative Neoplasms (MPNs) characterized with excessive erythropoiesis and thrombosis. Anoikis is a special programmed cell death mode induced by the adhesion disorder between cells and extracellular matrix (ECM) or adjacent cells facilitating cancer metastasis. However, few studies have focused on the role of anoikis in PV, especially on the development of PV.

**Methods:** The microarray and RNA-seq results were screened from the Gene Expression Omnibus (GEO) database and the anoikis-related genes (ARGs) were downloaded from Genecards. The functional enrichment analysis of intersecting differentially expressed genes (DEGs) and protein-protein interaction (PPI) network analysis were performed to discover hub genes. The hub genes expression was tested in the training (GSE136335) and validation cohort (GSE145802), and RT-qPCR was performed to verify the gene expression in PV mice.

**Results:** In the training GSE136335, a total of 1,195 DEGs was obtained from Myeloproliferative Neoplasm (MPN) patients compared with controls, among which 58 were anoikis-related DEGs. The significant enrichment of the apoptosis and cell adhesion pathways (i.e., cadherin binding) were shown in functional enrichment analysis. The PPI network was conducted to identify top five hub genes (CASP3, CYCS, HIF1A, IL1B, MCL1). The expression of CASP3 and IL1B were significantly upregulated both in validation cohort and PV mice and downregulated after treatment, suggesting that CASP3 and IL1B could be important indicators for disease surveillance.

**Conclusion:** Our research revealed a relationship between anoikis and PV for the first time by combined analysis of gene level, protein interaction and functional enrichment, allowing novel insights into mechanisms of PV. Moreover, CASP3 and IL1B may become promising indicators of PV development and treatment.

## Introduction

Myeloproliferative Neoplasms (MPNs) are the abnormal clonal myeloproliferation arising from the hematopoietic stem cells with acquired somatic mutation, and constitutional systematic phenotypes (Cervantes et al., 2008; Mead and Mullally, 2017). The latest World Health Organization (WHO) classified MPNs into chronic myeloid leukemia (CML) *BCR::ABL1* ^*+*^, polycythemia vera (PV), essential thrombocythemia (ET), and primary myelofibrosis (PMF) (Arber et al., 2016). The latter three are generally called *BCR::ABL1* ^*-*^ MPNs (hereinafter referred to as MPNs).

PV, the commonest MPN, is characterized with erythrocytosis in consequence of JAK2 exon 14 or 12 somatic mutations (Baxter et al., 2005; James et al., 2005; Kralovics et al., 2005; Levine et al., 2005; Scott et al., 2007). Unlike ET or PMF, PV is characterized by progressive increase in erythropoiesis, splenomegaly due to extramedullary hematopoiesis, and arterial and venous thrombosis (Spivak, 2017). In addition, some patients with PV are at risk of transformation to bone marrow failure, myelofibrosis, and acute leukemia (Spivak, 2017). Ruxolitinib, a typical JAK1/2 inhibitor, was used to provide systemic symptom relief in post-PV myelofibrosis (PPMF) (Verstovsek et al., 2012) and chronic-phase PV (Vannucchi et al., 2015; Passamonti et al., 2017), while did not promote apoptosis of hematopoietic stem cells (HSCs) and failed to achieve molecular remissions (Wang et al., 2014).

Anoikis, a type of programmed apoptosis, is induced by incorrect attachment between cells and extracellular matrix (ECM) or adjacent cells, playing important role in eradicating misplaced or dislodged cells to maintain tissue homeostasis (Han et al., 2021). Some studies suggested anoikis occurred through intrinsic/extrinsic pathways and could be triggered by several intracellular signals such as DNA damage and endoplasmic reticulum stress (Li et al., 2019; Zhi et al., 2022). Anoikis is essential for cancer cell survival through the detachment from ECM, and anoikis resistance is a key factor in cancer invasion and metastasis (Berezovskaya et al., 2005; Kim et al., 2012; Kakavandi et al., 2018; Xiao et al., 2019; Yu et al., 2022; Zhou et al., 2022).

Clonal cell proliferation leads to increased risk of thrombosis and threatens the life of PV and ET patients (Barbui et al., 2013). Whereas, complications of thrombosis in myelofibrosis (MF) are as frequent as in ET but less than in PV (Kc et al., 2017). Recently, the abnormal expression of adhesion molecules like *Lu/BCAM* and *CD147* was reported in PV patients, which was reduced under interferon-α treatment but enhanced by hydroxyurea (Brusson et al., 2018). These results indicated the important role of cell adhesion in PV and PV treatment. However, few studies had systematically evaluated the effect of anoikis in hematological cancers especially MPNs.

Therefore, in this study, we focused on exploring the expression levels of anoikis-related genes in the occurrence, progression and treatment of MPNs, as well as exploring the key anoikis-related genes by means of bioinformatics analysis.

## Materials and methods

### Data collection

Key word “myeloproliferative neoplasms” was used to search gene expression profiles of MPNs in the Gene Expression Omnibus (GEO, http://www.ncbi.nlm.nih.gov/geo/) data portal. The following criteria were used to filter the obtained database: 1. The gene expression profiling must cover all types of chronic and progressed MPNs (PV, ET, PMF) and controls. 2. The cell used for sequencing should be CD34^+^ MNCs (mononuclear cell) from peripheral blood (PB) or bone marrow (BM). 3. The processed data or raw data from datasets must be provided thus could be available to reanalyze. 4. The GEO dataset numbered GSE136335, GSE145802 were selected. Further, anoikis-related genes (ARGs) were downloaded from the GeneCard database (https://www.genecards.org/).

#### Design of this study

**FIGURE 1:**
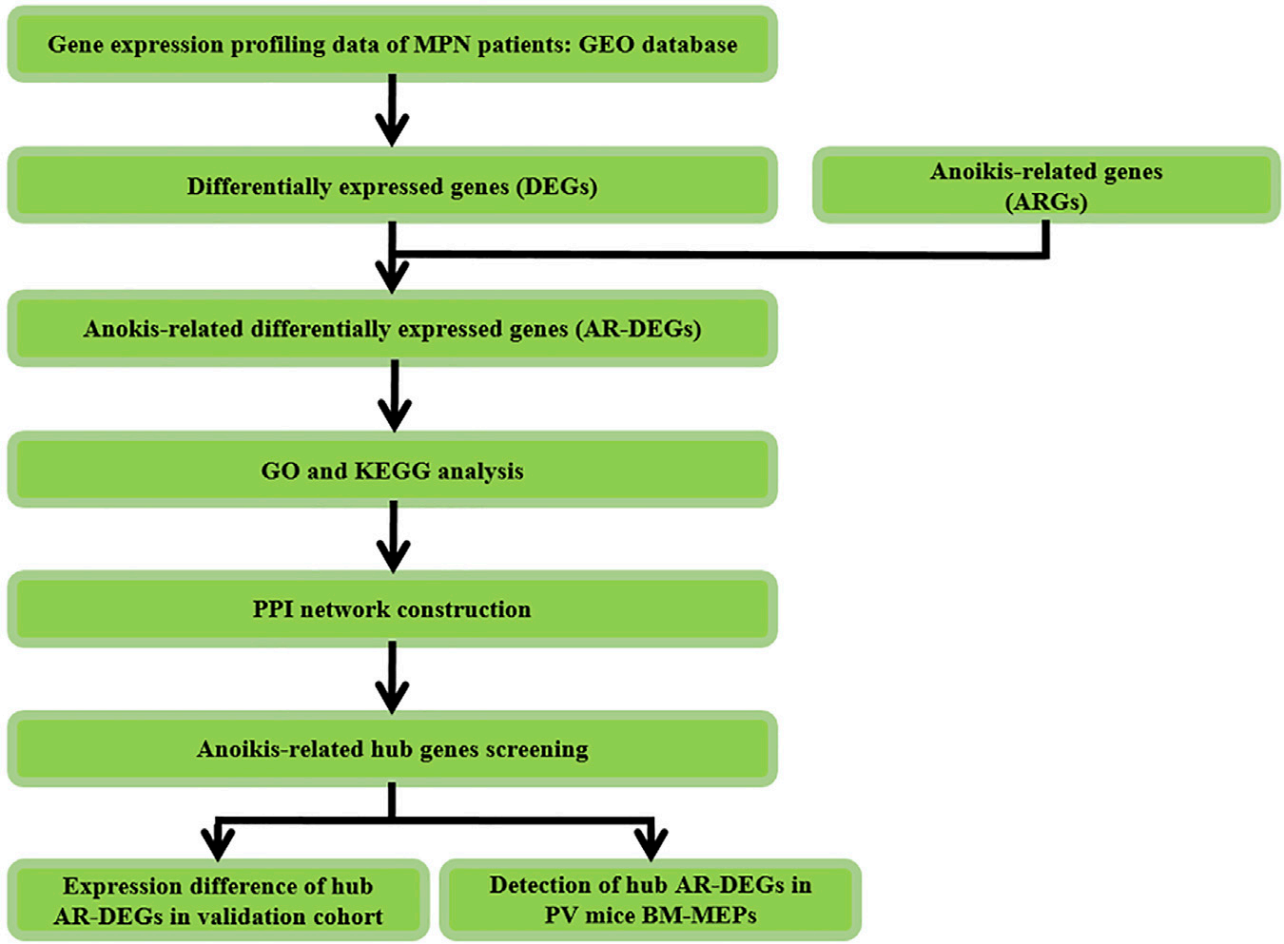
Study flowchart. GSE, Gene Expression Omnibus Series; GO, gene ontology; KEGG, Kyoto Encyclopedia of Genes and Genomes; PV, Polycythemia Vera; BM, Bone Marrow; MEPs, megakaryocyte and erythroid progenitors.

The study design was shown in Figure 1. Firstly, differentially expressed genes (DEGs) were screened in MPNs and MPN sub types including PV, ET, PMF and post-PV myelofibrosis (pPVMF), as well as the intersection of the DEGs and ARGs was analyzed. And then functional enrichment GO and KEGG analyses were performed. Lastly, PPI network analysis was conducted and the top 5 hub genes were found in the network. In addition, the corresponding hub genes in different validation cohorts were validated and the hub genes of PV in our mice model were detected.

### Data processing and screening of anoikis-related DEGs

Differentially expressed genes (DEGs) between patients with MPNs and healthy controls were identified in GSE136335 dataset *via* the “*limma*” R package (Gentleman, 2005), the cutoff for adjustment: *p*-value <0.05 and FC (fold change) > 1.5. The “*ggplot2*” R package (Ito and Murphy, 2013) was used to visualize the DEGs into a volcano map, while the “*pheatmap*” R package (Szekely and Rizzo, 2005) was used to cluster the anoikis-related DEGs. The intersection between DEGs and ARGs was visualized by the Veen plot.

Functional enrichment analysis and protein-protein interaction network analysis of anoikis-related DEGs.

Gene Ontology (GO) and Kyoto Encyclopedia of Genes and Genomes (KEGG) enrichment analyses of anoikis-related DEGs were performed by using “*clusterProfiler*” R package (Wu et al., 2021). GO analysis is important to explore the biological functions, consisting of three subtypes, including biological process (BP), cellular component (CC), and molecular function (MF) (Ashburner et al., 2000). KEGG analysis is used to explore potential pathways (Kanehisa and Goto, 2000) The STRING online website (https://string-db.org/) was to conduct the protein-protein interaction (PPI) network analysis of anoikis-related DEGs. The results of STRING were imported into *Cytoscape* (version 3.7.2), and the hub genes was extracted by using the *cytoHubba* plugin. Finally, top five genes were selected with the highest scores (*Degree* algorithm) in the network as hub genes.

#### Mouse model

Animal experiments were approved by the Central South University, Changsha, Institutional Animal Care and Use committee. Animals were maintained a 12:12 h light/dark cycle with a temperature-controlled specific pathogen free (SPF) environment. 8-wk-old male mice were used in this study. For analyses in the Jak2V617F bone marrow transplantation (BMT) model, experiments were performed in strict adherence to China laws for animal welfare. This study was approved by the Ethics Committee of The Second Xiangya Hospital of Central South University (20220247).

For gene expression analysis in the conditional Jak2V617F knock-in model of PV, bone marrow (BM) from primary Vavcre Jak2V617F (Vavcre^+^ Jak2^VF/+^) mice (Tiedt et al., 2008; Mullally et al., 2010) (Jackson Lab (United States) was transplanted into lethally irradiated C57BL/6 mice (Slac Laboratory Animal Inc. Shanghai, China) (PV group), and the recipients progressed to PV before being used in the MEPs (megakaryocyte and erythroid progenitors) flow sorting experiment. As competitor transplantation, bone marrow from C57BL/6 recipient mice was transplanted into lethally irradiated C57BL/6 recipients (WT group).

6–8 weeks after BMT, BM cells of PV and WT recipients were harvested from bilateral femurs and tibias by crushing bones with mortar and pestle using PBS (Servicebio). Cells were filtered through 70 μm nylon mesh to obtain a single-cell suspension. Red blood cells were depleted by using erythrocyte lysis buffer (ACK buffer, Invitrogen).

For FACS analysis and cell sorting: EasySep™ Mouse Hematopoietic Progenitor Cell Isolation Kit (Catalog #19856, STEMCELL) was used to enrich the Lin^−^ cells with biotinylated antibodies recognizing specific cell surface markers. The cell surface was stained using antibodies as shown in Supplementary Table S2: FITC-anti-CD4 (GK1.5, BD), FITC-anti-CD5 (53–7.3, Biolegend), FITC-anti-CD8 (53–6.72, Biolegend), FITC-anti-B220 (RA3-6B2, Biolegend), FITC-anti-TER-119 (Biolegend), FITC-Gr-1 (RB6-8C5, Biolegend), FITC-anti-Mac-1 (M1/70, Biolegend), PE-cy7-anti-Sca-1 (D7, BD), anti-PE-Kit (2B8, Biolegend), BV421-anti-CD34 (RAM34, BD), APC-Fcγ R III/II (93, Biolegend). Zombie aqua (Biolegend) was used to distinguish dead and alive cells. Live, singlet cells were selected for gating and cell sorting (Megakaryocyte Erythroid Progenitor cells, MEP: Lineage (CD4, CD5, CD8a, Mac1, B220,Ter119, Gr1)^−^ Sca1+in^−^ Sca-1^−^ c-Kit^+^ CD34^−^ Fcγ R III/II^−^ Zombie aqua^−^) (Mead et al., 2013; Rao et al., 2019). 80,000—120,000 MEPs were isolated from PV and WT recipient mice by FACS sorting and used to further gene expression analysis. Cells were analyzed on a Fortessa Flow Cytometer and sorted on a FACSAria-II cell sorter (BD biosciences). Data was analyzed either using FACS Diva software or FlowJo (version 10.8.1) software (Treestar).

### RNA extraction and quantitative real-time PCR

After extracting total RNA of flow-sorted MEP cells using TRIzol (Invitrogen, United States) according to the manufacturer’s protocol, reverse transcription high-capacity cDNA reverse transcription kit (AG, AG11728, Changsha, China) was performed. Lastly qRT-PCR using SYB Green Master quantitative PCR system (YEASEN, Shanghai, China) was conducted. Relative expression levels was calculated by using the 2^-△△CT^ method. The sequences of the primers were listed in Supplementary Table S3.

### Statistical analysis

R software (4.1.3) and GraphPad Prism 6.0 were used among all statistical analyses. Statistical significance was set at *p*-value <0.05 for all analyses.

## Results

### GEO information

The study flow chart was shown in Figure 1. The two GEO datasets numbered GSE136335 and GSE145802 were selected to study according to the criteria previously set. Information and characteristics of the three datasets were summarized in Table1. The GSE136335 was confirmed as a discovery cohort with GSE145802 paired as a validated cohort.

**TABLE 1:** Summary of GEO datasets involving MPN patients.

ID	GSE number	Platform	Samples	Source types	Group
1	GSE136335	GPL17586	9 PV patients	PBMC/BM CD34^+^ MNCs	Discovery cohort
			2 ET patients		
			6 PMF patients		
			2 pPVMF patients		
			6 controls		
2	GSE145802	GPL24676	17 PV patients	PBMC CD34^+^ MNCs	Validation cohort
			9 control		

GSE: Gene Set Enrichment; PB: Peripheral Blood; BM: Bone Marrow; MNCs: Mononuclear Cells.

#### Identification of anoikis-related DEGs between MPNs and controls

**FIGURE 2:**
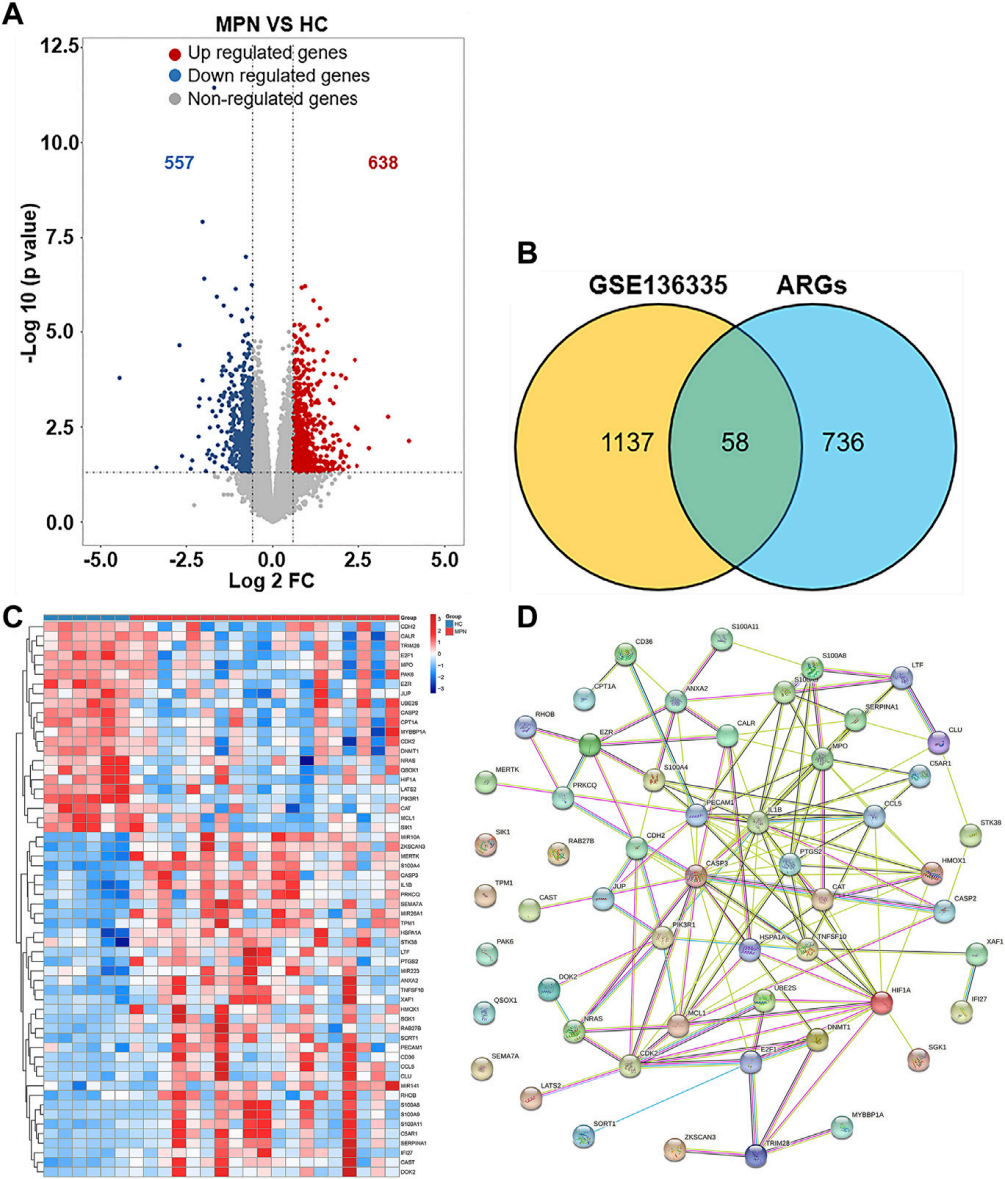
Screening of differentially expressed anoikis-related genes (ARGs) in MPN patients and healthy controls. **(A)** Volcano plot of genes differentially expressed between MPN patients and healthy controls in the GSE136335 dataset. Blue nodes represent downregulation in MPNs; red nodes represent upregulation; and grey nodes represent no significant difference from controls. **(B)** Intersection of differentially expressed genes (DEGs) in the GSE136335 and anoikis genes. The count on the left (1,137 genes) refers to DEGs unique to GSE136335; the count in the middle (58 genes) refers to anoikis-related DEGs; and the count on the right (736 genes) refers to unique to anoikis genes. **(C)** Heat map of 58 anoikis-related DEGs. **(D)** Protein–protein interaction (PPI) network of differentially expressed anoikis DEGs.

**FIGURE 3:**
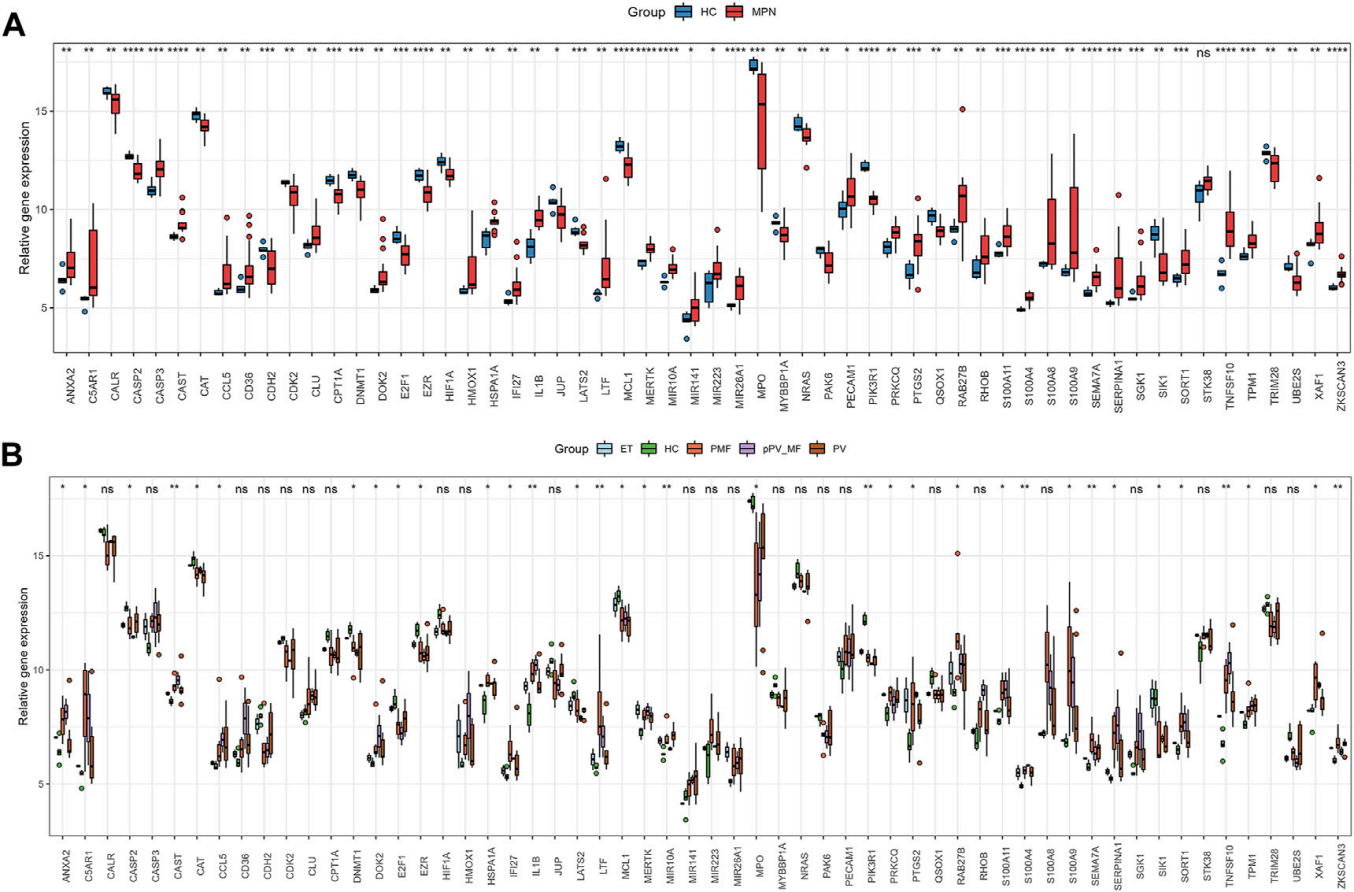
Expression of 58 anoikis-related DEGs in MPN **(A)** and subtype clusters **(B)**.

1195 DEGs were identified by screening GSE136335, with 557 downregulated genes (Figure 2A, blue dot) and 638 upregulated genes (Figure 2A, red dot). 795 anoikis-related genes were downloaded from the “GeneCard” database (Supplementary Table S1). Veen diagram (Figure 2B); the left region (1,137 genes) refered to unique DEGs in GSE136335; the middle intersection region (58 genes) refers to anoikis-related DEGs; and the right region (736 genes) refered to unique anoikis genes. The heat map in Figure 2C showed the standardized expression of anoikis-related DEGs (36 upregulated and 22 downregulated). The PPI network (Figure 2D) containing 54 nodes and 144 edges was obtained. Figure 3 showed the relative expression profiles of 58 anoikis-related DEGs in the MPNs (Figure 3A) and MPN-subtypes (Figure 3B).

**FIGURE 4:**
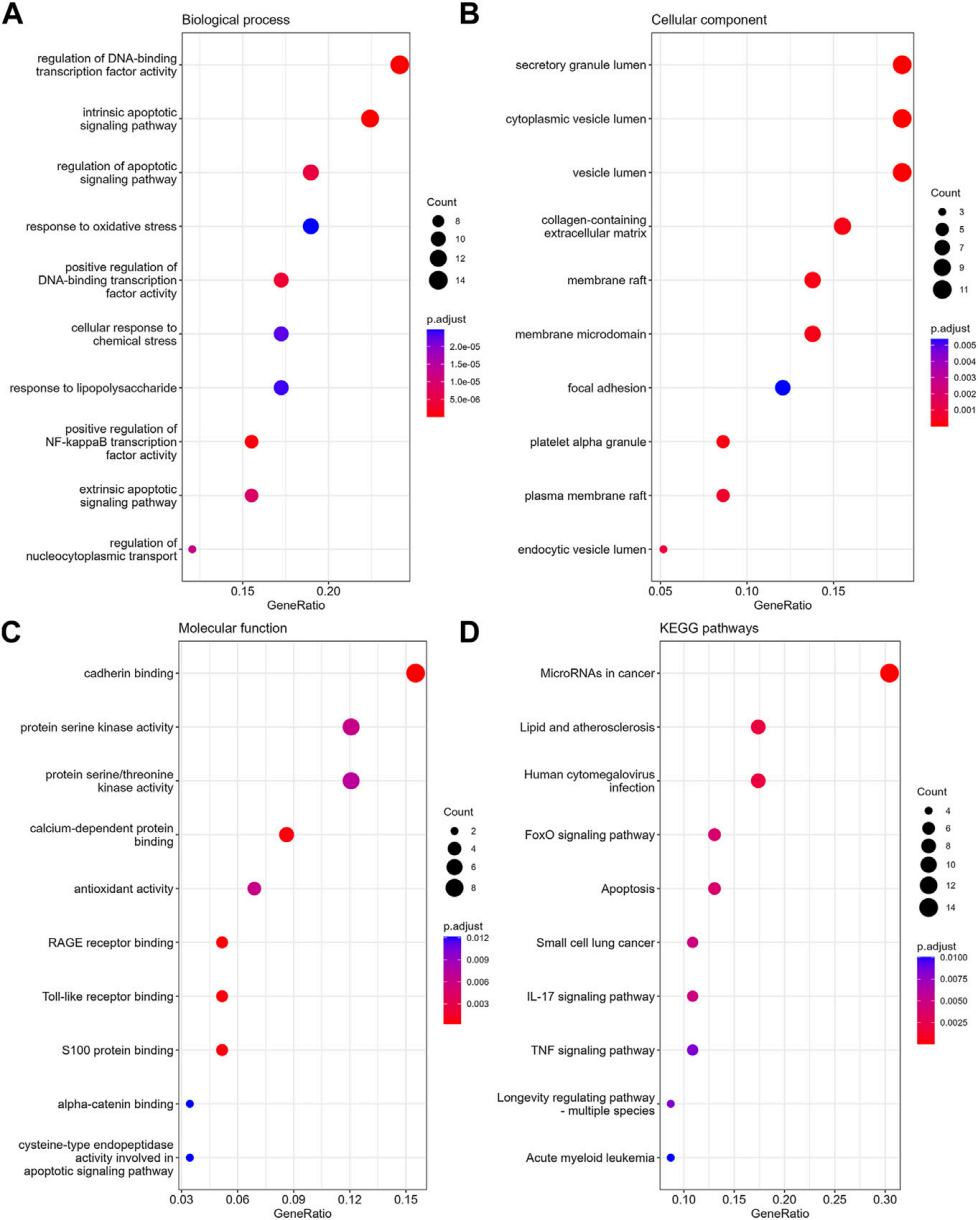
The enrichment analysis of anoikis-related DEGs in MPNs. The dot plots display the Top10 terms enriched by anoikis-related DEGs in BPs **(A)**, CCs **(B)**, MFs **(C)** and KEGG pathways **(D)**.

In GO-BP analysis (Figure 4A), the major pathways were also apoptosis or oxidative stress pathways. In GO-CC analysis (Figure 4B), collagen-containing extracellular matrix was significantly enriched. The results of enrichment analysis in GO-MF (Figure 4C) were mainly the activation of cell adhesion-related pathway like cadherin binding. In the KEGG analysis (Figure 4D), the top 10 enriched pathways were mainly apoptosis, FoxO signaling pathway, TNF signaling pathway, etc.

#### Identification of anoikis-related DEGs between PV and controls

**FIGURE 5:**
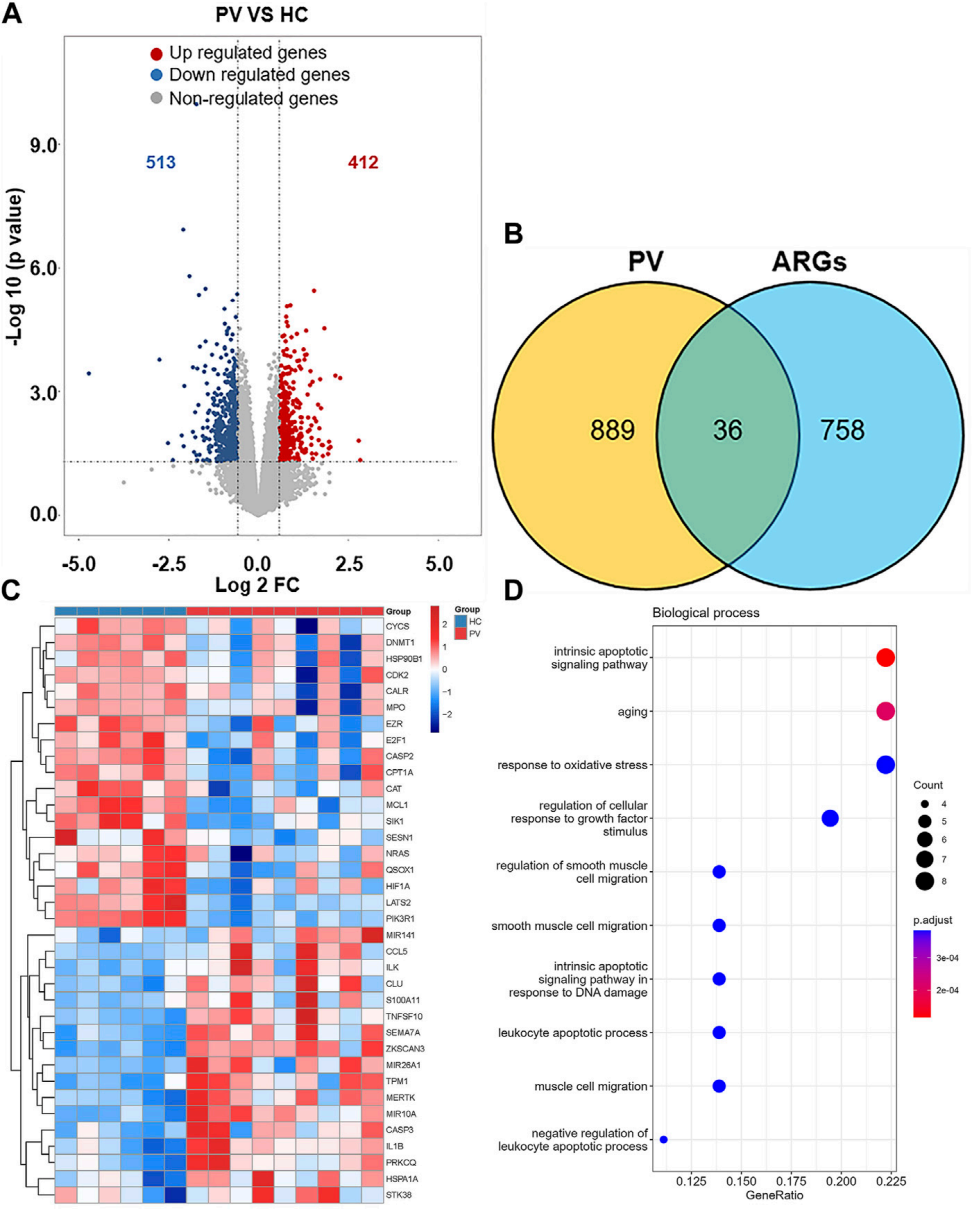
Landscape of Anoikis-related Genes in Polycythemia Vera. **(A)**Volcano plot of genes differentially expressed between Polycythemia Vera (PV) patients and healthy controls in the GSE136335 dataset. Blue nodes represent downregulation in PV; red nodes represent upregulation; and grey nodes represent no significant difference from controls. **(B)** Intersection of differentially expressed genes (DEGs) in the GSE136335 and anoikis genes. The count on the left (889 genes) refers to DEGs unique to GSE136335; the count in the middle (36 genes) refers to anoikis-related DEGs; and the count on the right (758 genes) refers to unique to anoikis genes. **(C)** Heat map of 36 anoikis-related DEGs. **(D)** Top 10 GO biological process pathway.

**FIGURE 6:**
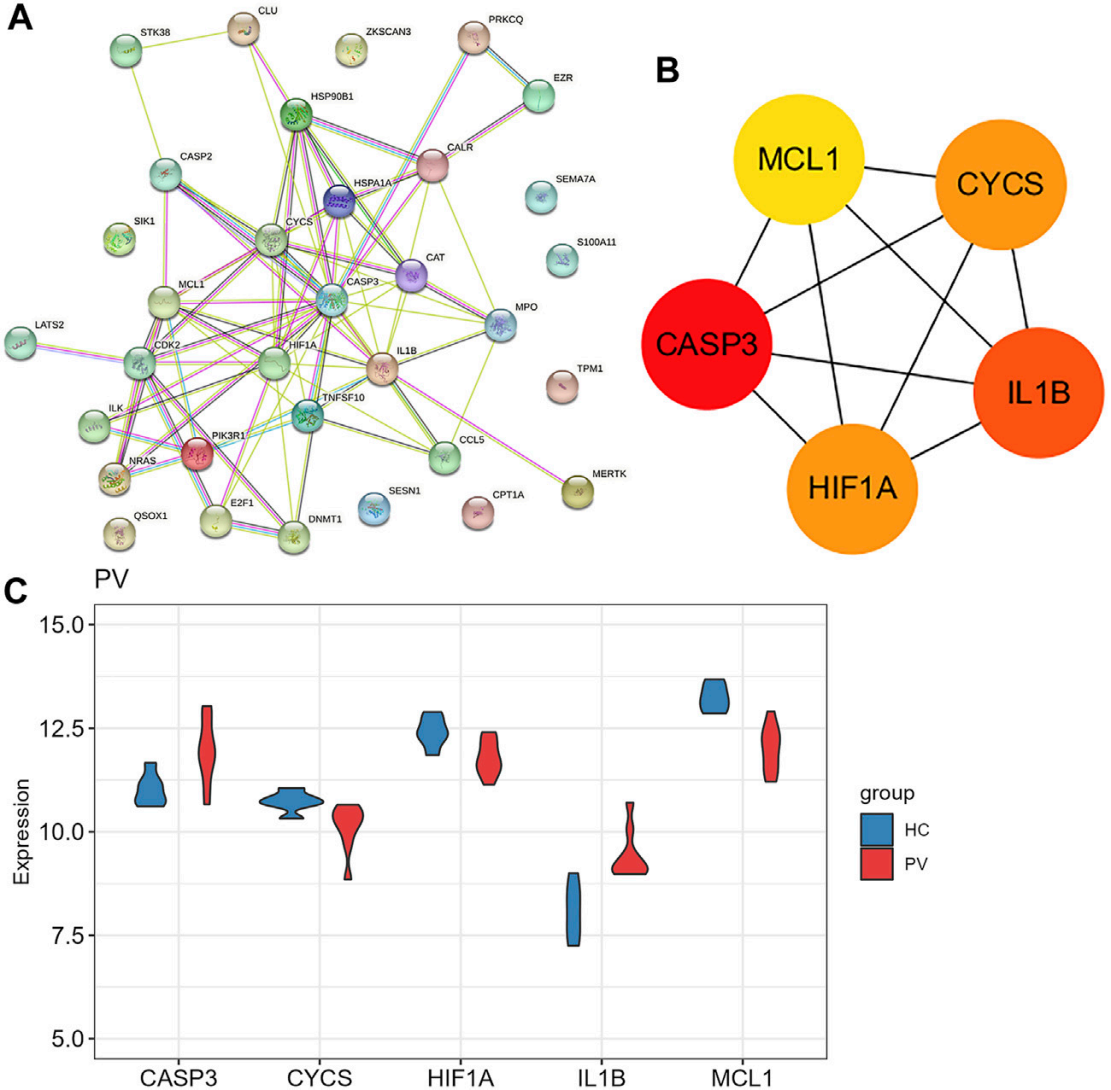
The protein-protein interaction network of anoikis-related DEGs in Polycythemia Vera. **(A)** PPI network for 36 anoikis-related DEGs; **(B)** Subnetwork of hub genes from the PPI network. Node color reflects the degree of connectivity (red color represents a higher degree, and yellow color represents a lower degree). **(C)** The expression difference of five hub genes in the training set (GSE136335).

925 DEGs were identified in PV patients compared with controls, with 513 downregulated genes and 412 upregulated genes (Figure 5A). Intersection analysis showed 36 anoikis-related DEGs (Figure 5B). The standardized expression of anoikis-related DEGs (17 upregulated and 19 downregulated) was shown in the heat map (Figure 5C). The results of enrichment analysis in GO and KEGG (Figure 5D; Supplementary Figure S1A) were also mainly focused on the apoptosis and activation of cell adhesion-related pathway like integrin binding. PPI network (Figure 6A) was constructed to select top 5 hub genes (*CASP3, IL1B, HIF1A, CYCS, MCL1*) (Figure 6B) according to the Degree algorithm. The expression levels of hub genes in training set were shown (Figure 6C).

**FIGURE 7:**
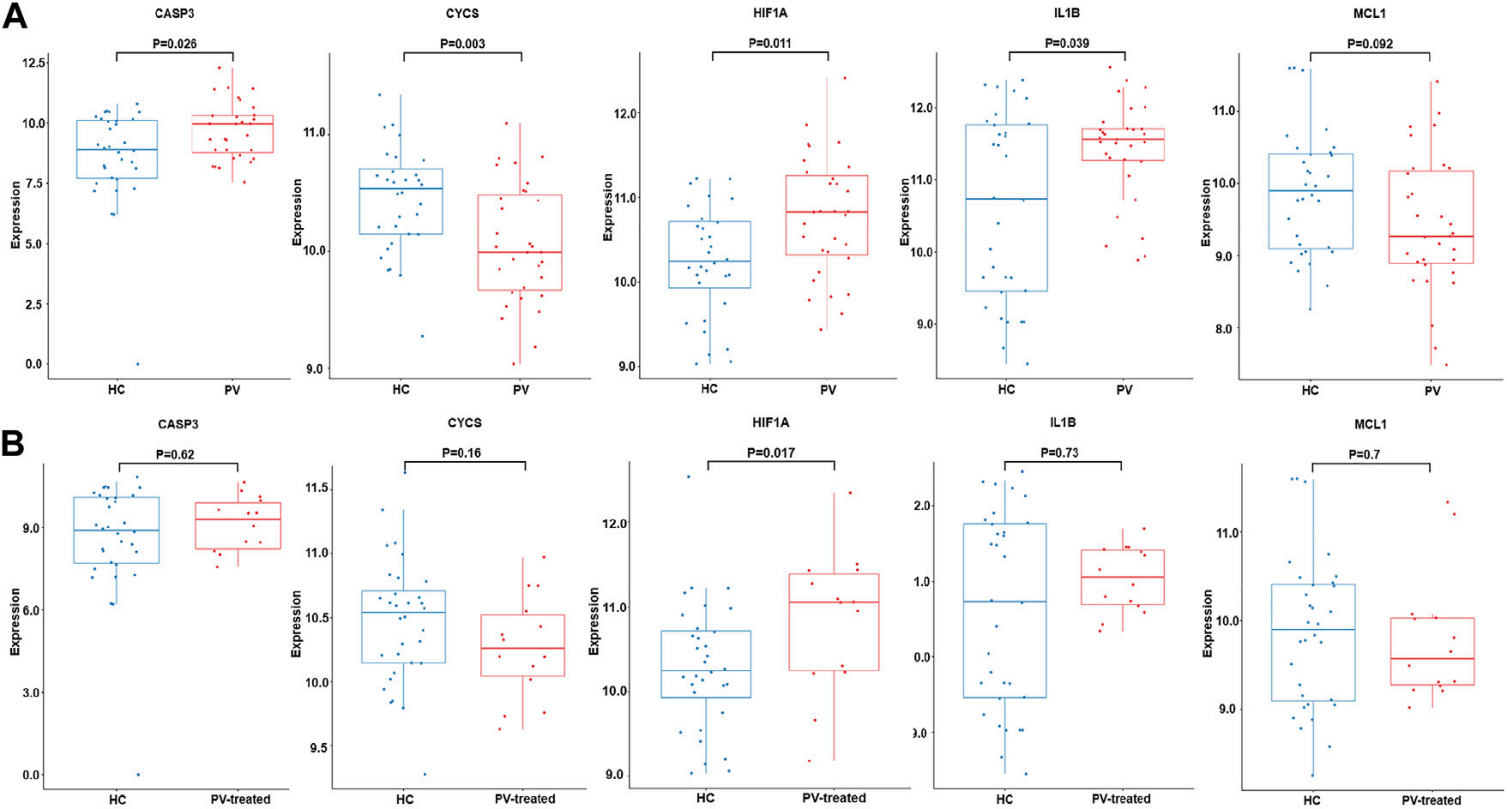
Expression of CASP3, CYCS, HIF1A, IL1B and MCL1. The expression difference of CASP3, CYCS, HIF1A, IL1B and MCL1 in GSE145802 validation set in untreated-PV patients (PV) and healthy controls (HC) **(A)**, and treated-PV patients (PV-treated) and HC **(B)**.

Validation analysis was conducted by using another dataset GSE145802. The expression levels of anoikis related DEGs in PV, *CASP3, IL1B, HIF1A* were significantly upregulated and *CYCS* was obviously downregulated, while the expression of *MCL1* was downward trend in PV patients compared with normal controls (Figure 7A). However, the expression levels of anoikis related DEGs in PV-treated, only *HIF1A* was upregulate (Figure 7B).

#### Expression difference of hub genes in PV mice model

**FIGURE 8:**
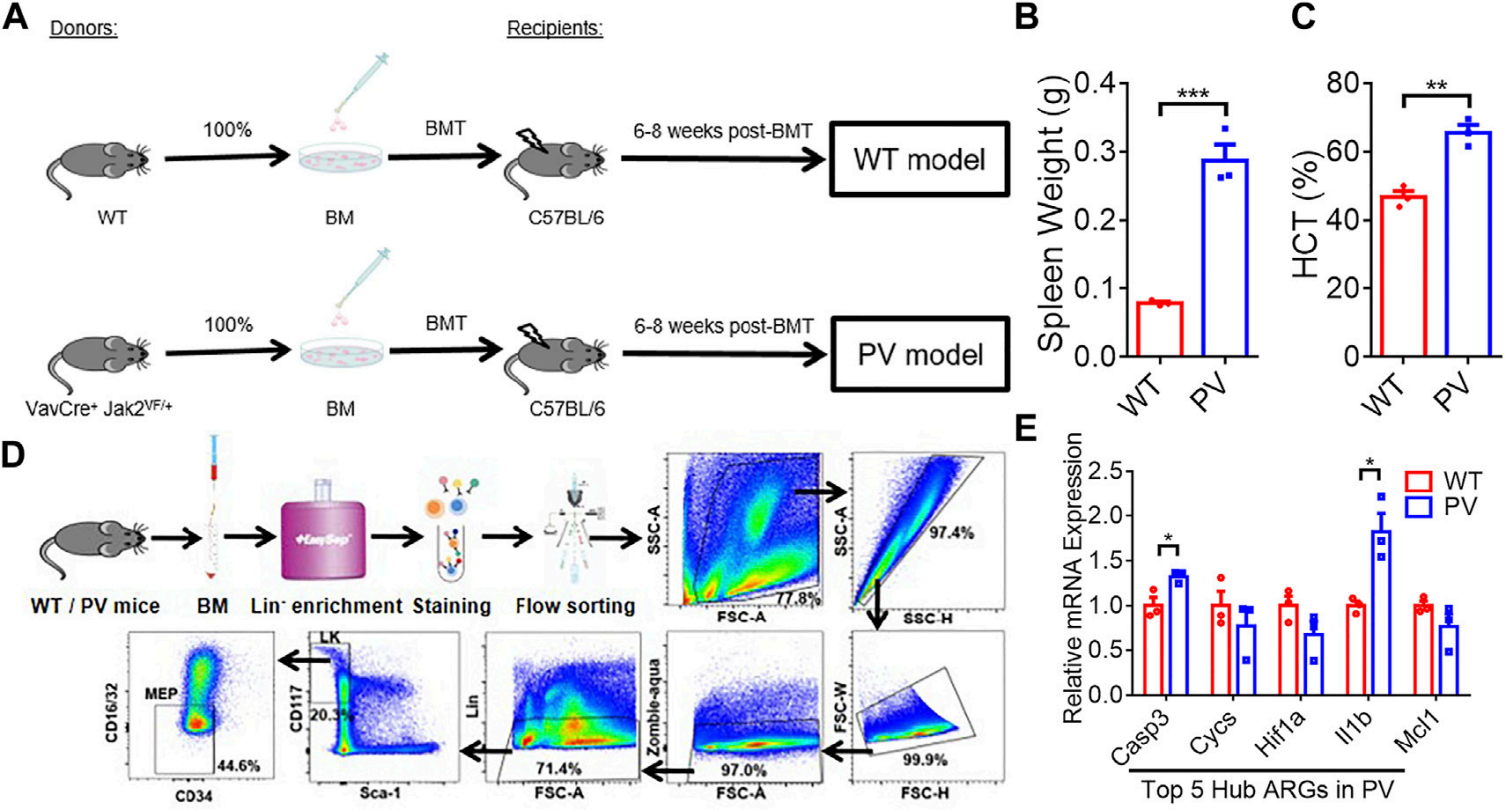
The expression difference of PV hub genes in PV mice model. **(A)** The workflow of WT and PV mice model constructed by bone marrow transplantation (BMT). **(B)** Spleen weight 6–8 weeks after BMT. Data are represented as mean ± SEM (***p < 0.001 versus WT group). **(C)** Hematocrit (HCT) 6–8 weeks after BMT. Data are represented as mean ± SEM (**p < 0.01 versus WT group). **(D)** The experimental procedure of obtaining WT and PV mice bone marrow megakaryocyte and erythroid progenitors (MEPs) by Lin-cell enrichment and flow sorting. **(E)** The validation of expression difference of five PV hub genes in WT and PV mice MEPs.

The expression of five PV hub genes was verified with our experimental data. PV mice model with Jak2V617F mutation (PV) and the corresponding WT mice (WT) were constructed by bone marrow transplantation (BMT) assay (Figure 8A). The spleen weight (Figure 8B) and hematocrit (HCT) (Figure 8C) of the recipient mice were measured 6–8 weeks after BMT, and the results indicated PV models successfully were constructed. PV and WT MEP cells were obtained by Mouse Hematopoietic Progenitor Cell Isolation Kit combined with FACS sorting (List of flow cytometry antibodies can be found in Supplementary Table S2) following the strict experimental procedures (Figure 8D). The relative mRNA expression (The sequences of the primers are listed in Supplementary Table S3) of Casp3 and Il1b were upregulated in MEPs of PV mice compared with WT mice (Figure 8E). However, other hub genes had no difference between these two group mice.

## Discussion

Myeloproliferative neoplasms (MPNs) are myeloid malignancies characterized by clonal expansion of one or more differentiated myeloid lineages (Dameshek, 1951). Anoikis, a particular form of programmed cell death, was reported to perform important biological function in preventing attachment or grow of dysplastic cell for tissue homeostasis and development (Bakir et al., 2020; Li et al., 2021). Anoikis plays a crucial role in tumor invasion, migration, distant metastasis and drug resistance (Jin et al., 2017; Jiang et al., 2020; Kim et al., 2021).

In our study, a new view of differential expression anoikis-related gene sets in MPN patients and controls was provided. MPNs are the malignancies caused by dysfunctional HSC, thus CD34^+^ PB/BM MNCs was chosen for bioinformatics analysis. The intersection of MPN-DEGs and ARGs revealed 58 anoikis-related genes were specifically associated with MPNs. Gene set enrichment analysis showed cell adhesion and apoptosis-related pathways were fully enriched to activate the anoikis pathway. However, in MPNs, circulating cells with disease driver mutations (such as JAK2V617F), including red cells, leukocytes, platelets, as well as some vascular endothelial cells, were abnormal (Hasselbalch et al., 2021). When activated in MPNs, these abnormal cells interacted with each other and created proadhesive and prothrombotic environment in the circulation to predispose patients to thrombosis and occlusive complications (Hasselbalch et al., 2021).

PV is characterized by mutations in JAK2 exon 14 or 12 in hematopoietic stem cells, leading to erythrocytosis and systematic symptoms (Greenfield et al., 2021). PV mainly reduces the quality of life and shortens the life span of patients due to the risk of vascular events and transformation to myelofibrosis, myelodysplastic syndrome, and acute myeloid leukemia (Tefferi et al., 2013; Tiribelli et al., 2015; Frederiksen et al., 2019). Our results found that 36 DEGs in PV were associated with anoikis. Combined with the results of gene expression and gene enrichment, CD34^+^ mononuclear cells in PV deflected stronger adhesion effect than apoptosis, in accordance with the characteristics of PV prone to thromboembolism. Five hub genes (*CASP3, IL1B, HIF1A, CYCS, MCL1*) was selected with highest score through PPI network combined further analysis. The pro-apoptotic gene *CASP3* was expressed higher in PV-CD34^+^ cells in the training and validation cohorts and in MEP cells in our PV mouse model, contrary to previous report (Steidl et al., 2005). The large variation may root in PV patients of different sources. The level of proinflammatory factor *IL1B* was upregulated in PV-CD34^+^ cells in training and validation cohorts and in MEP cells in our PV mouse model, consistent with previous report (Vaidya et al., 2012). Baumeister, Julian et al. reported higher protein level of HIF1α in 32D Jak2V617F-expressing cells than Jak2WT, and identified HIF-1 target genes involved in the Warburg effect as a possible underlying mechanism in JAK2V617F-positive MPN (Baumeister et al., 2020). The mRNA level of *HIF1A* was higher in PV-CD34^+^ cells in validation cohort. *MCL1*, a typical anti-apoptotic gene, plays a crucial role in the survival of HSCs and early progenitors (Opferman et al., 2005). Importantly, depletion of Mcl-1 was able to disrupt the viability of JAK2V617F mutant cells and sensitized mutant cells to JAK2 inhibitor therapy (Rubert et al., 2011). CYCS, as an apoptotic protein, is located between the inner and outer mitochondrial membranes (Ow et al., 2008; Shakeri et al., 2017) and is released into the cytoplasm in response to cell death signals and activated by pro-apoptotic Bcl2 family proteins (Wolter et al., 1997; Kennedy et al., 1999). The expressions of apoptotic-related genes *MCL1* and *CYCS* decreased in the PV-CD34 + cells of the training and verification cohorts, while no difference was found in the PV mice MEP cells. *CASP3* and *IL1B* were significantly increased in PV and decreased after treatment in validation cohort and expected to become indicators in PV development and treatment.

The inconformity between our results and previously reported may arise from following aspects: Firstly, the analyzed data was from peripheral blood or bone marrow mononuclear cells of MPN patients, excluding erythrocytes and granulocytes. Secondly, some vascular endothelial cells with driver mutations were also excluded. Thirdly, MPN in our study was a group of diseases rather than a single disease, and the degree of cell adhesion and apoptosis varies among subtypes. Finally, our results suggested that CD34^+^ mononuclear cells in bone marrow or peripheral blood contributed significantly less to thromboembolism than other types of cells.

Although MPNs are a myeloid proliferative disease, our research firstly revealed the critical role of anoikis-related genes in MPNs. *CASP3*, *CYCS*, *HIF1A*, *IL1B*, *MCL1* are the anoikis-related hub genes in PV and *CASP3* and *IL1B* may become important indicators of PV development and treatment. And our research may provide new insight into mechanisms of PV.

## Data Availability

The original contributions presented in the study are included in the article/Supplementary Material. Further inquiries can be directed to the corresponding author.
