# Supplementary material for: Exploration and identification of anoikis-related genes in polycythemia vera
Source: Front Genet. 2023 Feb 17;14:1139351. doi: 10.3389/fgene.2023.1139351 (PMC9981965; doi:10.3389/fgene.2023.1139351)
Supplement: Supplementary file 1 [file Presentation1.PPTX]

## Slide 1
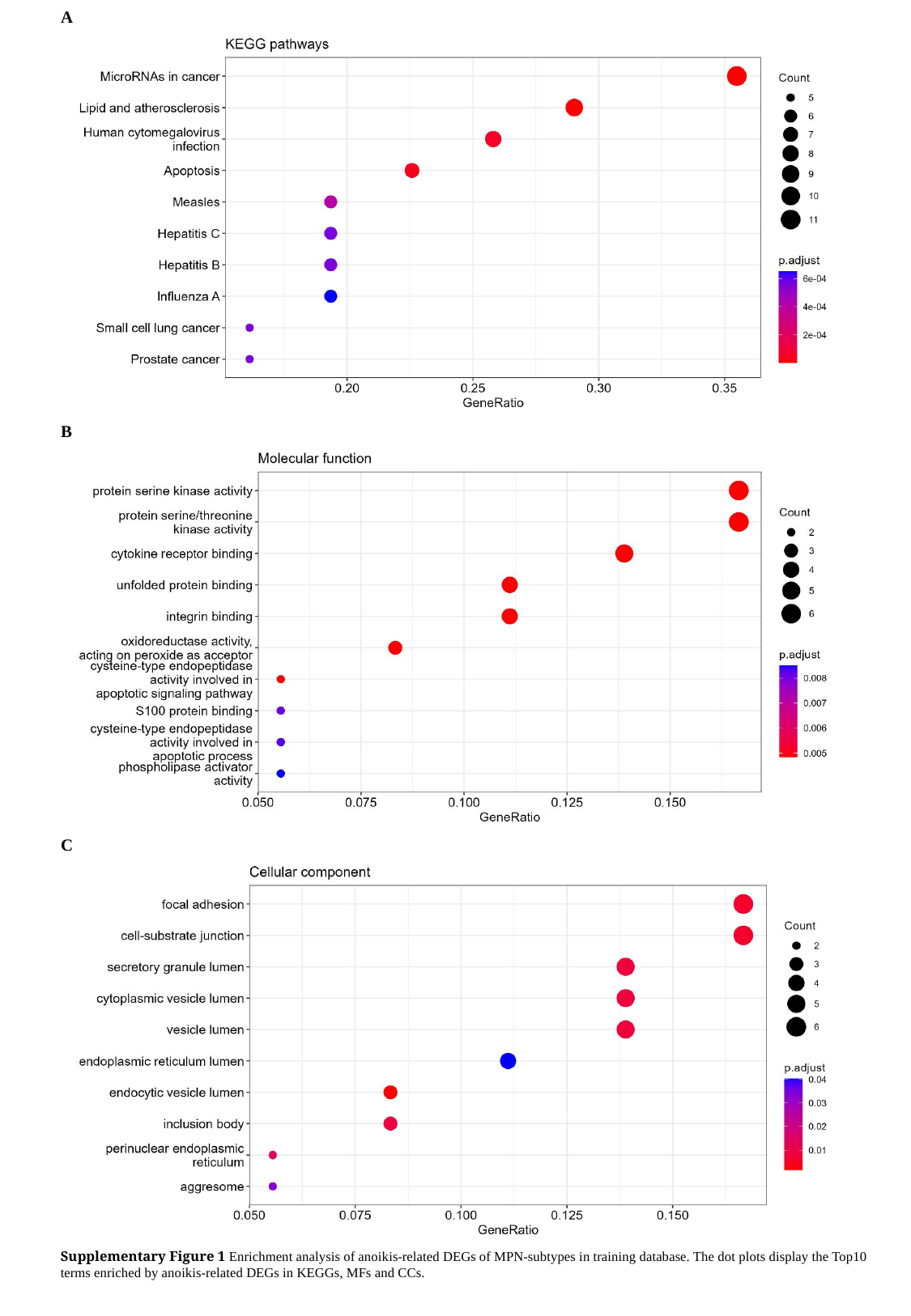

A
B
C
Supplementary Figure 1 Enrichment analysis of anoikis-related DEGs of MPN-subtypes in training database. The dot plots display the Top10 terms enriched by anoikis-related DEGs in KEGGs, MFs and CCs.
